# Wearable Activity Monitoring in Day-to-Day Stroke Care: A Promising Tool but Not Widely Used

**DOI:** 10.3390/s21124066

**Published:** 2021-06-12

**Authors:** Hanneke E. M. Braakhuis, Johannes B. J. Bussmann, Gerard M. Ribbers, Monique A. M. Berger

**Affiliations:** 1 Department of Rehabilitation Medicine, Erasmus MC University Medical Center, P.O. Box 2040, 3000 CA Rotterdam, The Netherlands; j.b.j.bussmann@erasmusmc.nl (J.B.J.B.); g.ribbers@erasmusmc.nl (G.M.R.); 2 Faculty of Health, Nutrition and Sport, The Hague University of Applied Sciences, 2521 EN The Hague, The Netherlands; m.a.m.berger@hhs.nl; 3 Rijndam Rehabilitation, 3015 LJ Rotterdam, The Netherlands

**Keywords:** wearable technology, rehabilitation, stroke, implementation, physical therapy

## Abstract

Physical activity monitoring with wearable technology has the potential to support stroke rehabilitation. Little is known about how physical therapists use and value the use of wearable activity monitors. This cross-sectional study explores the use, perspectives, and barriers to wearable activity monitoring in day-to-day stroke care routines amongst physical therapists. Over 300 physical therapists in primary and geriatric care and rehabilitation centers in the Netherlands were invited to fill in an online survey that was developed based on previous studies and interviews with experts. In total, 103 complete surveys were analyzed. Out of the 103 surveys, 27% of the respondents were already using activity monitoring. Of the suggested treatment purposes of activity monitoring, 86% were perceived as useful by more than 55% of the therapists. The most recognized barriers to clinical implementation were lack of skills and knowledge of patients (65%) and not knowing what brand and type of monitor to choose (54%). Of the non-users, 79% were willing to use it in the future. In conclusion, although the concept of remote activity monitoring was perceived as useful, it was not widely adopted by physical therapists involved in stroke care. To date, skills, beliefs, and attitudes of individual therapists determine the current use of wearable technology.

## 1. Introduction

Stroke is a major cause of disability and is an age-dependent problem [1]. With an aging society and improved acute care, the number of stroke survivors living with long-term stroke consequences is increasing beyond the level of increase of professional capacity [2,3]. Many stroke survivors show deteriorated levels of functioning, with low levels of physical activity [4,5]. Being physically active is an important determinant of social participation and is a major target of stroke rehabilitation [6]. Furthermore, being physically active is related to physical and psychosocial functioning, quality of life, and reduction of cardiovascular risk factors [7,8,9,10].

Physical activity is one of the components of physical behavior, that covers all movements, postures, and activities of a person’s during their daily life [11]. Another component is sedentary behavior, which is associated with cardiovascular disease incidence and mortality and depressive symptoms too [12,13]. Targeting stroke rehabilitation by increasing physical activity and decreasing sedentary behaviors may help to suppress the burden of stroke.

Stroke rehabilitation could benefit from remote monitoring of physical behavior with wearable sensor technology [14]. The development of wearable activity monitors has rapidly evolved over the last decades in academic research and the consumer market [15,16]. They provide an objective insight into behavior in a non-invasive and continuous way and can be applied in the home environments as well as in in- and outpatient settings to patients and therapists [17]. In addition, increased patient involvement by providing feedback on physical activity may enhance compliance and stimulate self-management [18]. The objective insights also allow therapists to set tailored therapy goals, guide patients towards them, and evaluate progress [19,20].

Although the body of evidence of remote monitoring of physical activity is growing in academic research, its clinical implementation lags behind [21,22]. Adopting technologies in day-to-day care routines seems challenging for therapists, who are key players in adopting remote monitoring of physical activity [22], since it requires careful attention, precious time, sufficient organizational and technical infrastructure, and knowledge [23,24,25,26,27]. Studies indicate that physical therapists acknowledge the potential benefits and practical purposes of wearable activity monitoring in rehabilitation therapy [28,29,30]. However, so far these studies have applied individual interviews and small focus groups. To provide an extensive insight into the current uses and clues on how to push the clinical implementation of this technology in stroke care forward, a study with a wide group of physical therapists involved in stroke care is needed. Therefore, the current study aimed to explore the use, perspectives, and barriers to potential applications of wearable activity monitoring in day-to-day stroke care amongst physical therapists in the Netherlands.

## 2. Materials and Methods

### 2.1. Participants and Data Collection

This cross-sectional study used an online survey (LimeSurvey^®^) among physical therapists in the Netherlands involved in post-stroke rehabilitation. Therapists were included if they were involved in the treatment of at least one stroke patient in the last month in a rehabilitation center, geriatric care center, or in primary care in the Netherlands. Participants were invited by e-mail with a web link via contact persons of seven primary care stroke networks in the Netherlands and ten Dutch rehabilitation centers and via a newsletter of the special interest group “rehabilitation” of the Royal Dutch Society of Physical therapy (KNGF: Koninklijk Nederlands Genootschap voor Fysiotherapie). After three weeks, a reminder for filling in the questionnaire was sent. Surveys were filled in anonymously.

### 2.2. Survey Development

A research team of physical therapists, human movement scientists, and researchers developed the survey based on literature and interviews. The survey included questions on demographic and occupational characteristics. Literature was used to formulate questions on the following topics: innovativeness (multiple choice answers to the question on innovativeness were based on the descriptions of the adoption categories of Rogers [31]), health care technology, activity monitoring outcome measures, perceived usefulness, barriers, and willingness to use it in the future [15,16,27,29,32] (See Supplementary Materials for the complete survey). To measure the attitudes of the participants regarding these questions, a 5-point Likert scale was used [33]. Participants were also asked if they were familiar with activity monitoring, if they use it for tracking their own activities, and if they already use it in stroke care. If a participant answered “yes” to the question concerning use in stroke care, they were defined as a user, and otherwise as a non-user. The users received additional questions about the use in day-to-day practice. They were asked how long they have been applying it, for how many patients per week, for what purpose, and what outcome of physical behavior they were interested in. Additionally, with an open-ended question, the reason for use was questioned. At the end of the survey, all participants were asked by an open-ended question if they wanted to share anything else on activity monitoring in stroke care.

To ensure common understanding, definitions were explained in between the questions (see Supplementary Materials). Experts and physical therapists checked the initial survey for face validity, comprehensibility, vocabulary, and layout. The survey was pilot-tested by five physical therapists in primary care before distribution.

### 2.3. Data Analysis

RStudio (version 1.2.50001, RStudio, Inc., Boston, MA, USA) was used for the data analyses. Descriptive analysis was provided for all questions with means (SD), frequencies, and percentages. The Likert package [34] was used to visualize the questions answered with a Likert scale. Differences between users and non-users were carried out with Chi^2^ and Mann–Whitney U tests. The significance was set at α = 0.05.

All individual answers to the open-ended question were collected in Microsoft Excel for qualitative analysis. All answers were divided into emergent themes. The most frequent, remarkable, or important issues that were relevant to this study were extracted and reported in the results.

## 3. Results

### 3.1. Participants

Over 300 physical therapists received the e-mail with the invitation to fill in the online questionnaire. Of them, approximately 100 therapists were recruited via a primary care stroke network and approximately 200 therapists were recruited via a contact person within their rehabilitation center. The survey was available from 1 March till 1 June 2020. n = 132 started the survey via the web link and n = 103 completed the questionnaire (78%). Only complete surveys were used for further analysis.

Table 1 shows the demographic characteristics of the participants. The mean age of the study sample was 42.2 (SD 12.1) years. Most of the participants worked in a rehabilitation center as a physical therapist (n = 58). Nine participants were employed in two or three different settings. All therapists were involved in the treatment of stroke patients. Other patient groups treated by the therapists were congenital and acquired brain injuries, (inactive) elderly, chronic diseases, orthopedic conditions, and sports injuries.

Twenty-seven percent used activity monitoring in the treatment of stroke patients and were defined as users. Characteristics of both groups and differences between them are presented in Table 1.

More than half of the non-users (59%) were familiar with activity monitoring before filling in this questionnaire. Similar percentages of users (54%) and non-users (53%) used a smartphone app or consumer-grade activity tracker for monitoring their own lifestyle and sports activities. Two participants (1.9%) considered themselves as people who were initially reluctant to use new healthcare technology and innovations. Most of the therapists in the total study sample described themselves as a person who had no problem going along with pioneers in healthcare technology and innovation but who did not initiate it themselves (60%). Only one (0.9%) of the therapists described himself as someone who invented and designed new healthcare technology and innovations and 18% of the total sample said they were someone who followed the latest developments in healthcare technology and innovation and looked for applications in practice.

**Figure 1:**
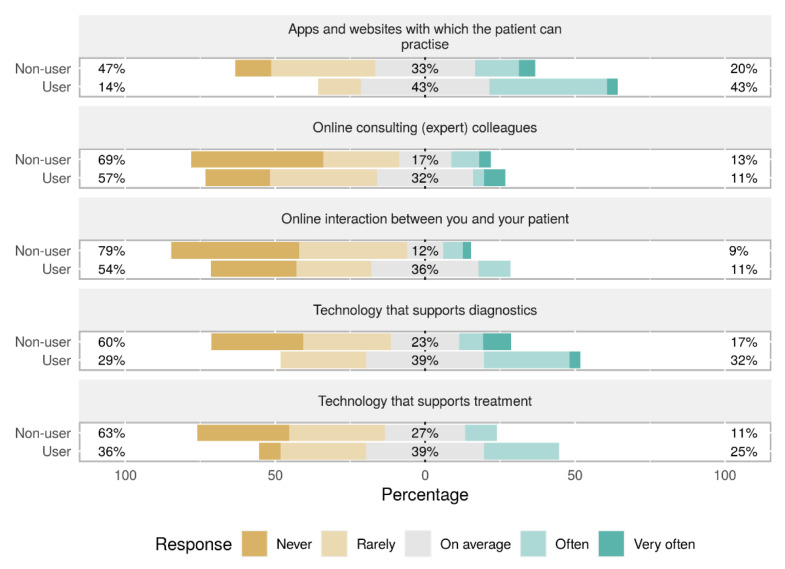
Other health technology used by participants, with differences between users and non-users.

The most often used health care technologies in the total study sample, other than activity monitoring, were applications and websites supporting the patient with practicing (21% often, 5% very often). The least often used was technology that supported diagnostics (15% often, 0% very often). Users of activity monitoring used significantly more other health care technologies (apps/websites, *p* = 0.036; online consulting (expert) colleagues, *p* = 0.023; technology that supports diagnostics, *p* = 0.009; and technology that supports treatment, *p* = 0.026) compared to non-users (Figure 1).

### 3.2. Users

Most users (54%) have been applying activity monitoring between six months and two years. Thirty-six percent have been applying activity monitoring shorter than six months, and eleven percent longer than two years. Most of the users applied activity monitoring between one and five patients per month (61%). Thirty-two percent applied activity monitoring in one patient per month or less, and seven percent in more than five patients per month.

**Figure 2:**
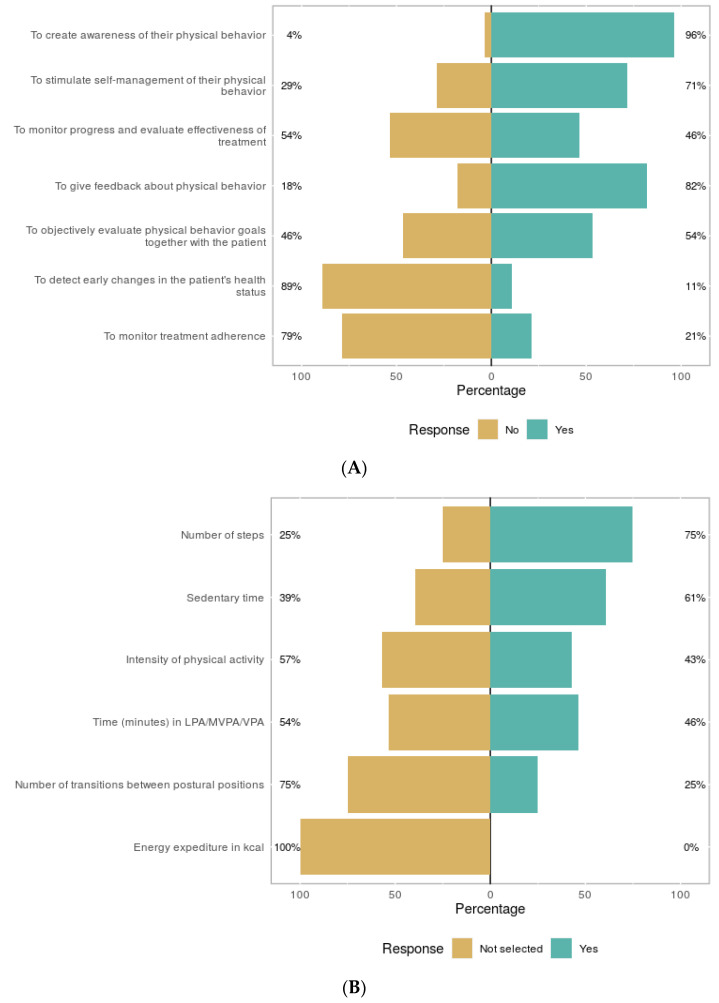
Treatment purposes (**A**) evaluated by users and outcome of interest of users (**B**). LPA= low physical activity, MVPA = moderate to vigorous physical activity, VPA = vigorous physical activity.

Figure 2A shows the treatment purposes of activity monitoring of the users. Almost all therapists used the monitor to create awareness for the patient with regard to their physical behavior (96%). Giving feedback about their physical behavior (82%) was also often recognized as a useful activity monitoring purpose. Figure 2B shows the activity monitor outcomes of interest during treatment. Most of them were interested in the number of steps. Additional outcomes of interest reported by users were heart rate and demands vs. capability, or in other words, the relation between what a patient did compared to what the patient was capable of.

In addition to the purposes in Figure 2A, users filled in for what reason they applied activity monitoring. Some of them reported new purposes compared to the ones provided in the answers; that they were instructed or motivated by external factors such as other colleagues who were already working with activity monitors or research/projects initiated by their organizations.

### 3.3. Perceived Usefulness

**Figure 3:**
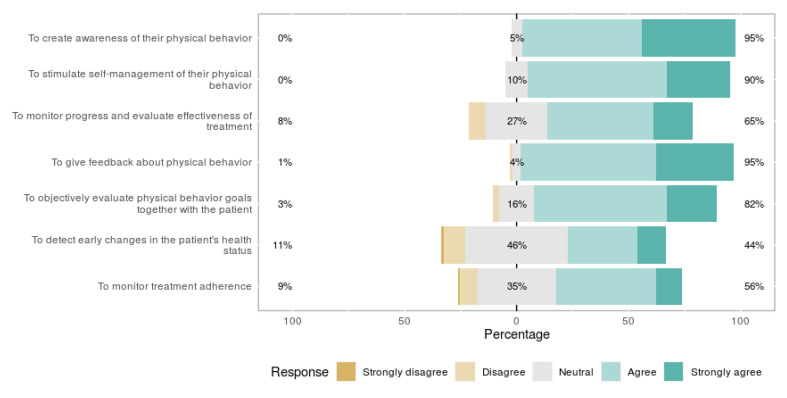
Perceived usefulness for eight different activity monitor purposes.

All participants (users and non-users) were asked for their opinion about the usefulness of activity monitoring for stroke patients. Six out of seven suggested purposes were considered useful by more than half of the study sample (Figure 3).

One significant difference was found between users and non-users: the users perceived creating awareness as more useful than non-users (*p* = 0.031). The participants were asked if they could come up with useful purposes other than noted in the question. Sixteen participants (16%) filled in the open-ended question on useful purposes other than mentioned in the question (Figure 3). Providing insight into a patients’ demands vs. their capabilities (n = 6) was the most common purpose. Two mentioned heart rate and one mentioned arm/hand use.

### 3.4. Barriers

**Figure 4:**
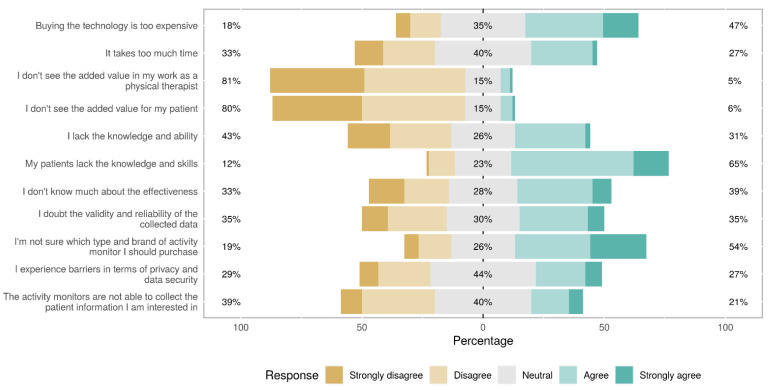
Barriers of using activity monitoring as a physical therapist.

The most present barriers reported by the whole sample were lack of skills and knowledge of patients (65%), not being sure what monitor to purchase (54%), finding it too expensive (47%), and taking too much time (27%). Overall, seeing no added value for their patients and their work as physical therapists was not recognized as a barrier by participants (Figure 4).

Non-users agreed more strongly with the following barriers compared to users: not knowing much about the effectiveness (*p* = 0.015), lacking knowledge and ability to apply the technology themselves (*p* = 0.013), finding it too expensive (*p* = 0.043), and not being sure what monitor to purchase (*p* = 0.035). Other barriers did not show significant differences between users and non-users.

### 3.5. Future Thoughts of Non-Users

Seventy-nine percent of the therapists who were not currently using activity monitoring were willing to use it in the future. Nineteen percent were neutral to this question, and two percent did not want to use activity monitoring in the future. In addition, participants were asked whether they would likely use activity monitoring in the next five years. Fifty-five percent of the non-users considered the change big or very big. Eight percent considered the change small or very small.

### 3.6. Additional Thoughts

The survey’s last question asked all participants if they wanted to share anything else on activity monitoring. Thirty-two participants (31%) filled in this question. Several positive and enthusiastic thoughts on activity monitoring were provided. Participants report that activity monitoring offers valuable insight into a patients’ behavior. About half of the 32 participants added some critical notes; they had doubts about the added value to the standard care relative to the effort. A few stated that applying technology was not always a holy grail and could not define therapy. Multiple participants mentioned that the usefulness was highly dependent on the age and stroke severity of the population.

## 4. Discussion

This study showed that, although physical therapists perceived wearable monitoring as potentially useful in stroke rehabilitation, only a minority of 25% actually used it in clinical care. Therapists that already used activity monitoring during treatment of stroke patients used it more often than other health care technologies and described themselves as being more innovative compared to non-users. The most recognized barriers were lack of knowledge and skills of patients, financial constraints, and not being sure what monitor to purchase.

The vast majority of our sample had not yet adopted the use of activity monitoring in day-to-day stroke care. The low numbers of technology used in treatment amongst physical therapists were in accordance with other studies that focused on technology use in rehabilitation practice [21,22]. A majority of 80% of therapists not using remote monitoring technology (non-users) did see value in the concept of objective physical behavior measurements with wearable technology, such as raising the patients’ awareness of their behavior and the ability of providing objective feedback in order to promote physical activity and were willing to use it in the future. Correspondingly, a majority disagreed with seeing no added value for their work as a therapist and for their patients as a barrier. Other studies also found positive attitudes and excitement of therapists towards the concept of objective physical behavior data collection in clinical practice [28,35].

The discrepancy between the levels of adoption of activity monitoring and its perceived potential value suggests the presence of barriers. Potential barriers to adoption were indeed identified. The most frequently recognized barrier (65%) was perceived lack of skills and knowledge to use wearable monitoring technology in patients. Obviously, cognitive problems and generally older age might complicate the use of technological devices in daily life in stroke patients [36]. Especially for this group of patients, a user-friendly design of technology is desirable [14,28]. Issues with older and more severely affected patients were also explicitly stressed by the therapists in the open-ended questions. It should be noted that these results represent a perception of the therapists and are not confirmed by the patients themselves. Mercer et al. [37] found that older patients with chronic conditions also saw meaningful potential for wearable activity trackers but acknowledged that help from health professionals was desired to integrate the use in their daily life. In addition, caregivers who know the patient and his circumstances can play a crucial role in successful adoption [38,39]. Their support and encouragement might help patients to learn how to use wearable technology in their daily lives. To further improve the adoption of remote monitoring of physical behavior, collaboration with end-users, both therapists, patients, and their caregivers is to be recommended [28]. Whether the device matches the needs of end-users seems a critical factor for successful use [40].

Another frequently recognized barrier, especially by the non-users, is the lack of skills in selecting and using the appropriate wearable activity monitor suitable for the patient. This might be aggravated by the increasing amount of available consumer and research-grade wearable monitors and their different specifications [23,41]. Research-grade devices are generally accurate and reliable but are not easy to use in clinical practice, whereas consumer-grade devices have limited accuracy in rehabilitation populations [23]. A clear overview of best practices and skill training for therapists may help to overcome this barrier. The non-users also expressed significant doubts about the effectiveness of wearable monitoring for stroke patients’ treatment. The field of research on the effectiveness for stroke patients is still evolving, more high-quality evidence might be a positive stimulus for use in the future [40,42]. Another critical concern physical therapists shared in the open-ended questions was that using technology can not define the course of therapy. Using technology should address the clinical need and the interaction between a patient and professional should not be forgotten [40].

Next to the individual skills and knowledge, successful, sustainable, and widespread adoption of technology is likely to be dependent on beliefs and attitudes of health care professionals [25,43,44]. Only one percent of the therapists in our study explicitly indicated being a person designing health care technologies and only 18% indicate that they are up-to-date and are looking for ways to adopt technology in daily practice. This low or absent innovative attitude might hamper the wide adoption in clinical practice. Therefore, if it is not widely accepted and fully integrated within organizations or the health care system, the use of wearable monitors will depend on the individual professional. Other stakeholders that have the potential to support and facilitate wider adoption of wearable technology are, for example, the policymakers of health care organizations, activity monitor companies, educational programs, and post-graduate training of professionals.

Our study has some limitations. As common in electronic surveys [21,27], non-response bias might have influenced our results. Respondents were probably more interested in contributing to a study on innovative technology than non-respondents, which may have overestimated the results. Since our respondents were selected based on being a physical therapist involved in stroke care, caution against generalizing our results to other health care occupations and patient populations is at its place. In addition, generalizability to other countries is limited since health care can be organized in a different way. We do not expect that geographical differences within the Netherlands have influenced our results since we tried to attempt diverse regions. No validated questionnaire that met our study purpose was available in the literature, and therefore to the best of our knowledge, we developed a survey with experts from the field and based on sufficient previous literature. The survey was pilot-tested amongst therapists and showed to be understandable and feasible. In addition, due to our study’s narrative and exploratory nature, we could not establish in-depth and underlying thoughts regarding the use of wearable technology for stroke patients. From our results, no extensive requirements or (sensor) features of wearable monitors for clinical practice could be derived. Future studies should provoke a more profound discussion with therapists about the need and requirements for wearable monitors and relevant datasets for clinical use. However, together with qualitative studies [28,29], our study contributed to a comprehensive understanding of physical therapists’ perspectives who, in the present years, are key stakeholders in adopting wearable technology in stroke care.

## 5. Conclusions

Our explorative study showed that despite physical behavior monitoring with wearable technology becoming commonplace in the consumer market and in academic research, it is not widely used by physical therapists involved in treatment of stroke patients. The concept of quantifying physical behavior with wearable monitors was perceived as useful by therapists, however, several barriers were identified. In current stroke care, physical therapists’ skills, beliefs, and attitudes determine the current use of wearable technology.

## Figures and Tables

**Table 1:** Demographic characteristics of respondents.

		Total (n = 103)	Users (n = 28) (27%)	Non-Users (n = 75) (73%)	*p*-Value
Age, mean (SD)		42.2 (12.06)	41.70 (13.24)	45.30 (12.11)	0.212
Gender (m/f)		26/76	8/20	18/56	0.420
Years of work Experience, n (%)	<5	9 (8.7%)	2 (7.1%)	7 (9.3%)	0.331
	5–10	18 (17.5%)	8 (28.6%)	10 (13.3%)	
	10–15	22 (21.4%)	7 (25.0%)	15 (20.0%)	
	15–20	7 (6.8%)	2 (7.1%)	5 (6.7%)	
	>20 years	47 (45.6%)	9 (32.1%)	38 (50.7%)	
Setting ^a^ (n)	Primary care	34	7	27	
	Rehabilitation	59	21	38	
	Geriatric care	20	2	18	

^a^ = participants were allowed to fill in multiple answers; user is defined by answering “yes” on the question if they already use activity monitoring during their work as a physical therapist.

## Data Availability

The data presented in this study are available on request from the corresponding author. The data are not publicly available.

## Supplementary Materials

The following are available online at https://www.mdpi.com/article/10.3390/s21124066/s1, Questionnaire wearable activity monitoring for physical therapists involved in stroke care.
