# Supplementary material for: Wearable Activity Monitoring in Day-to-Day Stroke Care: A Promising Tool but Not Widely Used
Source: Sensors (Basel). 2021 Jun 12;21(12):4066. doi: 10.3390/s21124066 (PMC8231529; doi:10.3390/s21124066)
Supplement: Supplementary file 1 [file sensors-21-04066-s001.zip › sensors-1194490-supplementary.pdf]

sensors

MDPI

Communication

Wearable Activity Monitoring in Day-to-Day Stroke Care: A Promising Tool but Not Widely Used

Hanneke E. M. Braakhuis [1,2,3,*], Johannes B. J. Bussmann [1,3], Gerard M. Ribbers [1,3] and Monique A. M. Berger [2]

1 Department of Rehabilitation Medicine, Erasmus MC University Medical Center, P.O. Box 2040, 3000 CA Rotterdam, The Netherlands; j.b.j.bussmann@erasmusmc.nl (J.B.J.B.); g.ribbers@erasmusmc.nl (G.M.R.)
2 Faculty of Health, Nutrition and Sport, The Hague University of Applied Sciences, 2521 EN The Hague, The Netherlands; m.a.m.berger@hhs.nl
3 Rijndam Rehabilitation, 3015 LJ Rotterdam, The Netherlands
* Correspondence: h.braakhuis@erasmusmc.nl

Supplementary Material:

Questionnaire Wearable Activity Monitoring for Physical Therapists Involved in Stroke Care.

1. **What is your gender?**
   - Male
   - Female

2. **What is your age?**

   (open-ended question)

3. **For how many years have you been working as a (BIG-registered) physical therapist?**

- > 5 years
- 5-10 years
- 10-15 years
- 15-20 years
- < 20 years

4. **In which setting are you working as a physical therapist?**

- Primary health care
- Rehabilitation centre
- Nursing home
- Other… *(please complete)*

5. **Which patient group do you primarily work with?**

- Stroke patients
- Other congenital and non-congenital neurologic conditions

- (inactive) elderly
- Patients with chronic diseases
- Orthopedic conditions
- Sports injuries
- Other.. *(please complete)*

6. **What is your highest level of education in the field of physiotherapy or another relevant field?**

- Bachelor physical therapy
- Master manual, sports or geriatric physical therapy
- Master neurorehabilitation or musculoskeletal rehabilitation
- Master human movement sciences, health sciences or comparable
- Master innovation in health care
- PhD
- Other .. *(please complete)*

The next two questions are about healthcare technology, also known as E-health, in general. Healthcare technology is a collective term for all digital and electronic means that help us to improve healthcare.

7. **When it comes to new healthcare technology and innovations, I would describe myself as...**

- Someone who invents and designs new healthcare technology and innovations
- Someone who is at the forefront, someone who follows the latest developments in healthcare technology and innovation and looks for applications in practice
- Someone who has no problem going along with pioneers in healthcare technology and innovation, but someone who doesn't initiate it themselves
- Someone who needs some time to get used to new healthcare technology and innovations, but ultimately participates in their deployment
- Someone who is initially reluctant to use new healthcare technology and innovations

8. **Do you already use the following healthcare technologies in your work as a physical therapist?**

- Apps and websites with which the patient can practise and/or have more control over their condition
- Interaction between you and your patient, such as making appointments online, e-consults
- Consulting (expert) colleagues, for example via videocalling or applications such as Siilo
- Technology that supports diagnostics, such as the use of applications to perform a gait analysis or to measure the range of motion
- Technology that supports treatment, such as exercises at home, monitoring and measuring instruments with sensors

The following questions are about a specific part of healthcare technology; objective measuring, also known as 'tracking', patients' exercise behavior by means of activity monitors. By exercise we mean all physical activities, but also the sedentary behavior of patients. Activity monitors, also called 'wearables' or 'activity trackers', are very promising tools for physiotherapy because they give you and your patient an objective insight into, for example, how much, how often and how intensively a patient exercises. In addition, the activity monitors also offer the possibility of automatically providing feedback on physical behavior. Examples of activity monitors are a Fitbit, the AppleWatch, but also step counter apps on your smartphone.

9. **Were you familiar with activity monitors as described above prior to taking this survey?**

- Yes
- No

10. **Do you use activity monitors to measure your own exercise behavior, such as sports activities, in your daily life?**

- Yes, …. *(please fill what you use)*
- No

The following questions are about the use of activity monitors during your work as a physical therapist. Although you may treat various patient groups, the questions below are specifically about the application of activity monitors in the treatment of stroke patients.

11. **Are you already using activity monitors as part of the treatment of your patients?**

** QUESTIONS 12 TO 17 WERE ASKED ONLY TO PARTICIPANTS WHO ANSWERED "YES" TO QUESTION 11 **

12. **For how long have you been applying activity monitoring as part of the treatment?**

- < 6 months
- 6 months – 2 years
- > 2 years

13. **For how many patients do you apply activity monitoring as part of the treatment on a weekly basis?**

- < 1 patient per month
- 1-5 patients per month
- > 5 per month

14. **What outcomes of physical behavior of your patients are you interested in?**

- Number of steps

- Sedentary time
- Intensity of physical activity
- Time (minutes) in low, moderate or vigorous physical activity
- Number of transitions between postural positions
- Energy expenditure in kcal
- Other .. *(please complete)*

15. For what treatment purposes do you use activity monitoring?

- To create awareness for the patient with regard to their physical behavior
- To stimulate the patient to take control of their own physical behavior
- To monitor the progress in the physical behavior of patients and thereby evaluate the effectiveness of the treatment
- To give the patient objective feedback about their physical behavior
- To objectively evaluate physical behavior goals together with the patient
- To detect early changes in the patient's health status
- To monitor treatment adherence
- Other purposes, namely...

16. What prompted you to apply activity monitoring?

(open-ended question)

17. To what extent do you think activity monitoring can be used for the treatment purposes mentioned above within physiotherapy?

Per treatment purpose:
5-point Likert scale: 1 = strongly disagree, 2 = disagree, 3 = neutral, 4 = agree, 5 = strongly agree

- To create awareness for the patient with regard to their physical behavior
- To stimulate the patient to take control of their own physical behavior
- To monitor the progress in the physical behavior of patients and thereby evaluate the effectiveness of the treatment
- To give the patient objective feedback about their physical behavior
- To objectively evaluate physical behavior goals together with the patient
- To detect early changes in the patient's health status
- To monitor treatment adherence

18. In addition to the treatment purposes mentioned above, are there any other purposes for which you think activity monitoring would be useful within physiotherapy?

(open-ended question)

19. What barriers have prevented you/could prevent you from using activity monitors in treatment?

Per barrier:

5-point Likert scale: 1 = strongly disagree, 2 = disagree, 3 = neutral, 4 = agree, 5 = strongly agree

- Buying the technology is too expensive
- It takes too much time
- I don't see the added value of using the technology in my work as a physical therapist
- I don't see the added value for my patient
- I lack the knowledge and ability necessary to apply such technology
- My patients lack the knowledge and skills necessary to use the technology
- I don't know much about the effectiveness of using activity monitors
- I doubt the validity and reliability of the collected data
- I'm not sure which type and brand of activity monitor I should purchase
- I experience barriers in terms of privacy and data security
- The activity monitors are not able to collect the patient information I am interested in

20. To what extent are you open to using activity monitors in your work as a physical therapist?

5-point Likert scale: 1 = Not at all, 2 = a little, 3 = neutral, 4 = open, 5 = very open

21. How likely do you think it is that you will use activity monitors in the next 5 years in your work as a physical therapist?

5-point Likert scale: 1 = very small, 2 = small, 3 = neutral, 4 = big, 5 = very big

22. Is there anything else you would like to mention about the use of activity monitoring as a physical therapist?

(open-ended question)
